# The Effect of the COVID-19 Pandemic on Pulmonary Tuberculosis Control in the Selected Upazila Health Complexes of Dhaka Division, Bangladesh

**DOI:** 10.3390/tropicalmed7110385

**Published:** 2022-11-17

**Authors:** Fariha Alam Mihika, Md Abdullah Al Jubayer Biswas, Md Maruf Haque Khan, Syed Shariful Islam, M. Atiqul Haque, Sayera Banu, Md Zakiul Hassan

**Affiliations:** 1 Programme for Emerging Infections, Infectious Disease Division, International Centre for Diarrhoeal Disease Research, Bangladesh (icddr,b), Mohakhali, Dhaka 1212, Bangladesh; 2 Department of Public Health and Informatics, Bangabandhu Sheikh Mujib Medical University (BSMMU), Dhaka 1000, Bangladesh

**Keywords:** tuberculosis service, presumptive tuberculosis, COVID-19, Bangladesh, Upazila Health Complexes, National Tuberculosis Control Program

## Abstract

Despite the enormous disruption of tuberculosis (TB) services reported globally, Bangladesh’s impact is not well documented. We aimed to assess the effect of the COVID-19 pandemic on the TB control program in Bangladesh from patients’ and service providers’ perspectives. We conducted a cross-sectional study from November–December 2021 at six conveniently selected Upazila Health Complexes (UHC) of the Dhaka division, Bangladesh. We conducted face-to-face interviews among 180 pulmonary TB service recipients and all TB service providers working in the selected UHC. We also reviewed TB registries from each UHC. All data were summarized using descriptive statistics tools. We found a 31% reduction in presumptive TB cases during 2021 compared to 2020. Other TB services, such as testing, were reduced by 16–36% during the same period. Service receivers reported a lack of transportation (95%), and a lack of adequate human resources (89%) as critical barriers to receiving and providing TB service, respectively. The findings of our study showed substantial interruption of TB service delivery during the COVID-19 pandemic, threatening the recent progress and pushback from achieving the 2035 End TB targets. Early mitigation of TB service delivery through adopting remote follow-ups using digital health technology and integrating COVID-19 and TB screening is essential for the continuity of essential TB services and achieving global TB targets.

## 1. Introduction

Globally, from the beginning of 2020, the coronavirus disease 2019 (COVID-19) pandemic has substantially sabotaged many aspects of health systems [1,2]. The response to the pandemic, which included measures such as a nationwide state of emergency, reorganization of medical staff, supplies, and diagnostic instruments, along with a decrease in outpatient services, had a considerable influence on the way many health services, including tuberculosis (TB) services, functioned and responded [3,4,5]. As a result, it is hardly unexpected that TB detection and reporting have fallen dramatically in the first few months of the epidemic, whether in developing or developed countries [6,7]. Subsequently, modeling-based research predicted that, over a three-month shutdown period, worldwide TB case detection would reduce by an average of 25% compared to pre-pandemic levels [8]. Likewise, an investigation of the first three months of the pandemic was conducted in 165 countries, and 42% reported partial interruptions in TB case diagnoses and management [9]. The findings of another study on 33 sites across 16 countries also revealed a similar reduction in hospital discharges related to TB, first-time suspects of TB cases, the total number of outpatient TB treatment visits, newly Latent Tuberculosis and bacterial infection (LTBI), and LTBI outpatient care visits [1].

Similar to other countries, the COVID-19 pandemic in Bangladesh has significantly affected the healthcare systems and socio-economic structures, including diminishing the number of persons seeking and getting TB-related health treatment [7,10,11]. Bangladesh has 3.6% of the world’s cases of TB, and TB patients are advised to be treated at a TB hospital or a Directly Observed Treatment Short-course (DOTs) clinic, the most efficient and long-lasting component of the National Tuberculosis Control Program (NTP) [12]. Nevertheless, the massive confinement policy and the suspension of public and private transportation during COVID-19 led to a severe breakdown in the surveillance, treatment, and prevention of tuberculosis [7]. According to the US Agency for International Development (USAID) TB Data Hub, there was an increase in all forms of TB cases during the last ten years in Bangladesh, which significantly declined and hit an all-time low point of 74,000 in 2021 [13]. Difficulty in accessing a DOTs clinic, the suspension of essential laboratory services and the inaccessibility of the few remaining open testing facilities were the causes of such disruptions [7].

Even though much has been published about the influence of COVID-19 on TB treatment services and occupational safety, little is known about the country-specific evaluation of presumptive TB (PTB) cases, registered adult PTB cases and laboratory activities, including sputum microscopy and gene Xpert machine use [7]. Despite the significant interruption of TB treatment during the COVID-19 pandemic in Bangladesh, the challenges and barriers experienced by service recipients and providers at DOTs clinic are not widely documented. Therefore, we aimed to compare TB service delivery in DOTs facilities during the COVID-19 period in Bangladesh with data from the previous year. In addition, we also aimed to identify the difficulties and challenges encountered by service recipients and providers at DOTs facilities during the pandemic. Our study’s findings will provide a comprehensive picture of the TB situation in Bangladesh, including diagnosis and access to healthcare facilities during this global health crisis. The results will also allow policymakers to better comprehend and plan future steps to address the limitations in combating TB during any pandemic situation, such as COVID-19, in Bangladesh, as well as reinforce the plan to improve the guidelines.

## 2. Materials and Methods

### 2.1. Study Design, Study Setting, and Study Participants

We conducted a cross-sectional study from 1 January to 31 December 2021. During the study period, the second wave of COVID-19 began in mid-March, and a rigorous lockdown was enforced on 14 April 2021 [14]. As a result, research efforts were severely hindered; this is why we purposefully selected six Upazila Health Complexes (UHC) from the Dhaka division to minimize transportation difficulties and maintain COVID-19-related precautions. Our chosen UHCs were the Sirajdikhan Upazila Complex, Munshigonj; Kaliganj Upazila Health Complex, Gazipur; Savar Upazila Health Complex, Savar; Singair and Harirampur Upazila Health Complex, Manikganj; and the Rupganj Upazila Health Complex, Narayanganj. Table 1 represents the detailed demographic data of the study Upazila health complex. We recruited study participants and collected primary data from adults with pulmonary TB who were about to finish their treatment or had already completed their treatment and healthcare providers engaged in TB treatment services at UHCs. Healthcare providers encompassed medical officers (MO), medical officer disease control (MODC) DOTs clinic service providers and laboratory staff working in the TB control service at those six selected UHCs. We exclude those TB service recipients who had extrapulmonary TB, were pregnant women, had emergency medical conditions, and were under 18 years.

### 2.2. Sample Size and Sampling Technique

We calculated the sample size by using the following formula [22]. Since no relevant research was found in Bangladesh, we relied on the proportion of TB patients in India who did not finish treatment due to the COVID-19 pandemic. According to their findings, seventeen percent of TB patients were not able to receive treatment due to the COVID-19 pandemic [23].

Sample size, n = [DEFF ∗ Np(1 − p)]/[(d2/Z21 − α/2 ∗ (N − 1) + p ∗ (1 − p)].

**Figure 1:**
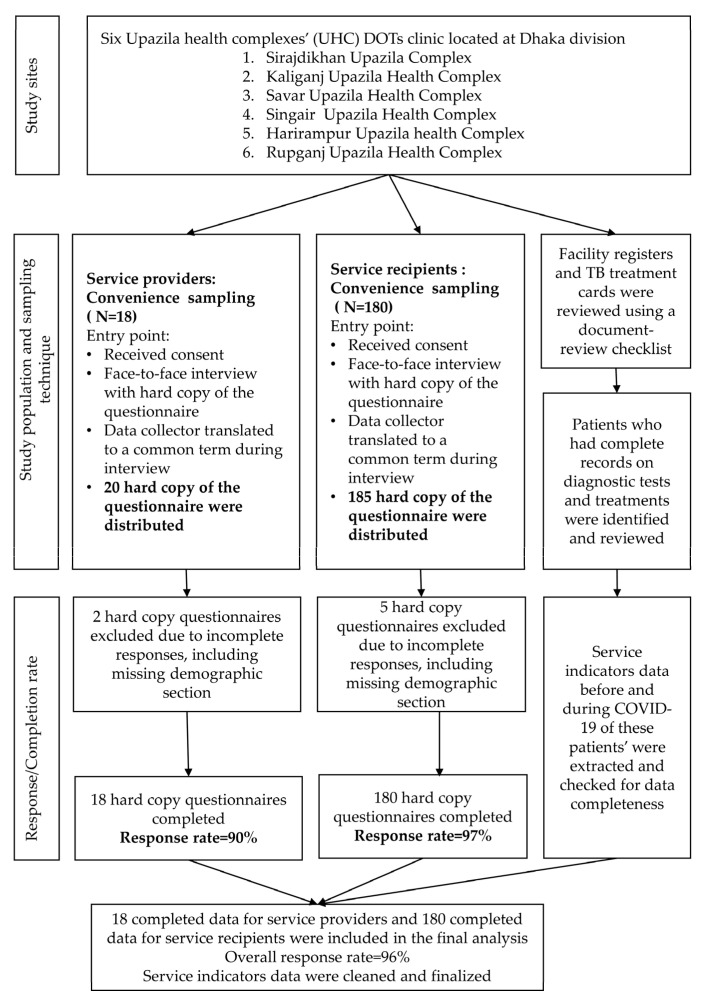
Flowchart depicting the data collection survey procedure.

Considering a 10,000 population size (N), 17% point estimate (p), 1% precision (d), 95% confidence interval (CI), 1 design effect (DEFE) and 20% non–response rate, the estimated sample size from the following formula is 178. We next allocated 180 recipients of pulmonary TB services recipients evenly across six UHCs, 30 from each UHC. Regarding recruiting service recipients, we utilized the non-probability convenience sampling technique considering the contemporary threat of COVID-19. Additionally, we selected all service providers working in the TB control program of each Upazila. There was one DOTs clinic in each Upazila, with an average of three service providers. In total, we enlisted 18 service providers from six Upazilas, three from each facility. Figure 1 depicts detail of the data collection survey procedure.

### 2.3. Data Collection Tools and Data Collection

We developed a semi-structured, standardized questionnaire following a comprehensive literature review [7,24,25,26,27,28]. We designed different survey questionnaires for service providers and service recipients. Additionally, we prepared a document-review checklist based on services offered by the DOTs corner to gather data from the DOTs corner’s TB registries. The questionnaires were pre-tested on five service providers and recipients and revised depending on their feedback. The final data set was prepared, excluding data gleaned during the pre-tested interviews. The survey questionnaire for service providers consisted of twenty questions separated into five sections: (a) demographic characteristics (seven questions); (b) obstacles and actions performed in the course of giving TB treatment (four questions); (c) COVID-19 screening of the TB patient (two questions); (d) adaptation in the DOTs clinic during COVID-19 (two questions); and (e) challenges in the management of TB patients (five questions). Similarly, the TB patient-related (service recipients) survey questionnaire had two sections with a total of seven questions, such as (a) socio-demographic (five questions), and (b) Access to healthcare for pulmonary TB patients during the COVID-19 pandemic (two questions) (Supplementary Materials S1). Service providers and receivers were interviewed face-to-face by trained data collectors. A team of field staff collected data. The field staff interviewed patients and providers, using a structured questionnaire to collect their data. The data collected included age, gender, designation duration of experiences, facing different challenges for providing or receiving service during the COVID-19 pandemic, different types of adaptation required in the DOTs clinic, etc. (Supplementary Materials S1). Field staff were also instructed to ensure that all participants provided written informed consent before conducting a face-to-face interview. During the consent process, participants were informed about the aims and objectives of the study, study procedure, benefits and risks of the participation, right to refuse to participate or withdraw from the survey, confidential data handling, and the principal investigator’s identity. The participants were also informed that anonymity and confidentiality were maintained by using identification numbers, and ensured that all data would be coded and stored in a locked cabinet. They were assured that the collected information would be used only for research purposes, and not otherwise.

In addition, the document review checklist consisted of TB service indicators, including the number of Presumptive TB patients, patients who tested sputum microscopy, registered adult PTB cases, and patients who tested by gene Xpert one year before COVID-19 and one year after the COVID-19 pandemic. We utilized the checklist to review TB registries from each UHC (Supplementary Materials S1). Figure 1 depicts the details of the study’s sampling technique and data collection survey process.

### 2.4. Data Analysis

We entered the collected data into MS Access, and then transferred it to the Statistical Package for Social Sciences (SPSS) version 23( International Business Machines Corporation, New York, United States). After this, we examined the data for incompletion or discrepancy due to inconsistent data, missing data and protocol deviations. Then, we identified the causes of the discrepancies, resolved them with supporting documentation, and updated the database. Before obtaining the final dataset, all variables were examined for inconsistencies, and all these problems were resolved. We used descriptive statistics tools to summarize all the variables, including frequency, percentage, mean, median, standard deviation (SD) and interquartile range (IQR), depending on the type and distribution of the variables. We computed the total number of patients in each service indicator from March 2020 to February 2021 (one year after COVID-19) and March 2019 to February 2020 (one year before COVID-19). All analysis was performed using SPSS 23.0.

### 2.5. Ethical Consideration

The Institutional Review Board of Bangabandhu Sheikh Mujib Medical University (BSMMU), Dhaka, Bangladesh, reviewed and approved the study protocol (BSMMU/2021/8366). The protocol received administrative endorsement from the National TB Control Programme, Director-General of Health Services, Mohakhali, Dhaka a. Study participants provided informed written consent.

## 3. Results

Table 2 shows the demographics of the survey participants. We found that among 180 service recipients, 67% were male, 66% lived in rural areas, and the mean age was 43 years (standard deviation 17). Regarding service providers, 83% were male, the mean age was 39 years, and the average experience was nine months. On average, three service providers were employed in each TB control service clinic, and fifty percent were infected with COVID-19 while on the job and took twelve days off due to the infection (Table 2).

### 3.1. Reduction of TB Service Indicators

We found that during the first year of the pandemic, the overall number of notifications of suspected presumptive TB patients and sputum microscopy tests were significantly reduced by 31% and 36%, respectively, compared to the previous year. Similarly, the total number of cases that tested positive by sputum microscopy and registered adult PTB cases reduced by 32% and 15%, respectively, but these were not statistically significant (Table 3).

**Figure 2:**
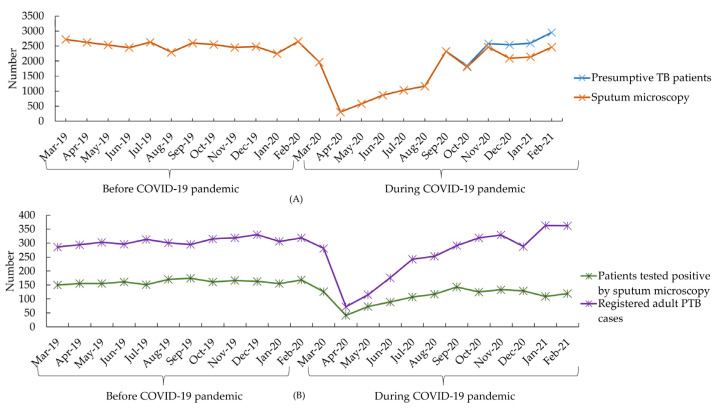
Monthly change in service delivery indicators from March 2019 to February 2021, Dhaka division, Bangladesh (**A**) monthly change in presumptive TB patients and sputum microscopy test (**B**) monthly change in registered adult PTB cases and patients testing positive by sputum microscopy.

We also found a sharp fall in the indicators during March 2020, and then all the indicators rose gradually (Figure 2). Specifically, from March 2019 to April 2020, the number of presumptive TB cases and sputum microscopy tests exhibited overlapping patterns, beginning with 2720 cases in March 2019, after a fall in the number seen in both cases. After April 2020, both indicators showed a similar upward trend, and ended with 2956 and 2462, respectively (Figure 2A). Likewise, the number of patients who tested positive by sputum microscopy and registered adult PTB cases showed a similar pattern, but the overall number of registered adult PTB cases was lower than test-positive patients throughout the study period. Both exhibited consistent trends from March 2019 to April 2020, followed by a dramatic decline, with each number hitting an all-time low of around 117 and 253 by April 2020. Between April 2020 and February 2021, both indicators’ numbers showed an upward trend (Figure 2B).

### 3.2. Challenges and Dissatisfaction among Service Recipients

**Figure 3:**
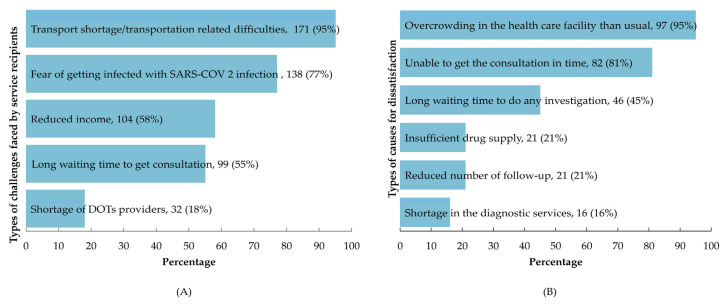
Different challenges and causes of dissatisfactions, 2021, Dhaka division, Bangladesh (**A**) type of challenges experienced by service receivers who received DOTs corner services during COVID-19 (N = 180; multiple responses) and (**B**) type of causes for dissatisfaction mentioned by service recipients who did not feel satisfied for any of the listed issues (N = 102; multiple responses).

We found that nearly all service recipients attended for treatment, follow-up and regular anti-TB medicine administration. Regarding the reported barriers while receiving care from DOTs, 95% of service receivers claimed difficulty in transportation, 77% asserted fear of getting infected and 58% mentioned a reduction of income (Figure 3). About half of the people who received services during the study period were dissatisfied. Of these, 95% complained about the facility being more crowded than usual, 81% said they couldn’t get a consultation in time, 45% complained about the length of time it took to conduct an investigation and 21% complained about the number of follow-up visits being reduced (Figure 3).

### 3.3. Barriers Faced and Adaptation Reported by the Service Providers

Among 18 service providers, almost 89% reported challenges in providing TB services in the DOTs clinic due to the COVID-19 pandemic. Among those who reported barriers, all said a lack of workforce and increased workload, 63% mentioned interruption in the regular follow-up of the patients (Table 4). Interestingly, all service providers reported adjustments required in the DOTs clinic. The most adaptation stated by the service providers were adaptation in behavior (94%), TB management (94%), TB screening management (94%), TB diagnosis (78%) and follow-up (44%). Likewise, all the service providers cited sample collection interruptions, patient non-compliance and canceled follow-up appointments as the most common challenges to diagnosing, treating and follow-up TB patients (Table 4).

## 4. Discussion

Our study revealed that the COVID-19 pandemic caused significant disruptions to TB service delivery. Overall, within a year of the onset of the COVID-19 pandemic, there was a substantial drop in the number of presumed TB patient notifications, undergoing sputum microscopy testing, sputum microscopy-positive cases and registered adult PTB cases. These findings highlight the need for urgent actions to ensure the continuity of TB care to achieve the global TB targets by 2035 set by the WHO End TB Strategy.

The reduction in PTB patient notification or registration in our study was comparable to the reports found in the early months of COVID-19 in several other TB-burdened countries, including Pakistan, Nepal and Malawi, where fear about contracting the virus and the temporary shutdown of healthcare facilities were reportedly the key factors [29,30,31,32]. Our results are also consistent with studies showing a drop in TB program activities of 67.3% in sputum microscopy tests in Nepal and 44.1% in recorded adult PTB cases in Malawi during the first months of COVID-19 compared to the same period in the previous year [32,33,34].

Additionally, our study found that a significant amount of service recipients reported difficulty in transportation, particularly the lack of available vehicles and/or the high cost of travel. Receiving treatment from DOTs clinics was also highlighted as a barrier due to fears of contracting an infection. During the pandemic, it is possible that people were afraid to go outside since the media constantly reported the latest fatalities. As a result of hearing about these deaths, individuals might have dreaded contracting COVID-19. Furthermore, TB and COVID-19 have many of the same symptoms, leading to increased stigmatization of those with TB who were concerned about contracting COVID-19 [28]. Our findings suggested that ensuring more accessible TB treatment and care services to patients with PTB may enhance service delivery activities during the pandemic, especially in TB notification and registration. Besides, awareness efforts may also be implemented among people with suspected cases of TB to alleviate the stigma and fear surrounding the current and potential future pandemics.

We found transportation as a key barrier in receiving TB services due to the countrywide lockdown, which is consistent to reports from other countries [35,36]. During lockdowns, all kinds of transportation remained halted, except those necessary for emergencies [35,36]. A study in India on TB patients reported transportation as a major barrier [37]. This study informed that transportation expenses were high during the lockdown period, notably in faraway places where mass transportation was unavailable [37].

Other than that, our survey identified overcrowding in the healthcare facility and the inability to obtain timely consultations. This was likely due to suspending regular treatment at many healthcare facilities’ DOTs clinics during the COVID-19 pandemic, which swamped the remaining operational TB clinics [7]. Thus, a strategic approach to facilitate transport or door-to-door serum collection during the pandemic can be adopted for TB patients who had trouble affording access to TB examination and out-of-pocket transportation charges.

Moreover, a lack of personnel, increased workload, stoppage in the routine follow-up of patients, interruption in the TB diagnosis and interruption in the medicine supply were cited as obstacles experienced by the TB services provider. Possible reasons for these issues include the redeployment of personnel from national TB programs, TB laboratories, and TB wards to the battle against COVID-19. A worldwide alliance of TB civic groups’ study found that TB treatment and research resources had been diverted to combating the newly emerged virus COVID-19 [38]. Our results are also supported by several studies from countries with a high Tb burden. In China, 75.2% of the country’s healthcare workers were reallocated to combat COVID-19 [39]. Likewise, in Pakistan, TB-dedicated laboratories were converted into additional COVID-19 testing centers [40]. Due to such reallocation and conversion, regular TB treatment services were significantly hampered; in South Korea, for instance, an estimated 2.3% of drug-susceptible PTB cases were lost to follow-up [41].

A potential solution to reallocate resources to minimize TB service disruption could be integrating COVID-19 and TB screening. Such adjustments in TB service during the COVID-19 pandemic have been reported in several countries, including India, Indonesia and South Africa. In India, state governments proposed implementing door-to-door TB screening programs and ordering District TB officers to examine COVID-negative individuals for TB [42,43]. In South Africa and Indonesia, strategies for screening for both TB and COVID-19 in both directions were outlined by the National TB Elimination Program (NTEP) [44,45].

This study had several strengths. Firstly, we gathered real-time data on regular monthly services from six different UHC DOTs corner registries to investigate interruption of service indications. Next, we compared the logged information with the records kept by monitoring and evaluation personnel; therefore, the data are more reliable and accurate. Secondly, we compared data across two consecutive 12-month periods, enabling us to account for any seasonal fluctuation that could have affected access to health facilities, such as during the rainy season. However, our analysis had several limitations. Due to data collection challenges during the ongoing pandemic, we only include subdistrict hospitals from one of seven administrative divisions (e.g, Dhaka division). Hence, our results may not represent another part of the country not studied. However, the structure and facilities are similar across all subdistrict hospitals, and the reported impact of such lockdown would be similar across Bangladesh. Lastly, while HIV status is a key factor in determining TB risk, we could not assess HIV status, as testing facilities are not widely available, including in our study hospitals. Future studies should incorporate assessing the HIV status of TB cases as recommended by the Nation TB control program guidelines [46].

## 5. Conclusions

The COVID-19 pandemic caused a substantial decrease in TB service delivery, including case detections and testing threatening the recent progress and pushback from achieving the 2035 End TB targets. Our studies highlighted several challenges faced by the TB service receiver and service provider. Some of these challenges included a lack of transportation, a lack of adequate human resources, and logistics. Mitigating these challenges through adopting remote follow-up using digital health technology and integrating COVID-19 and TB screening would be important for maintaining essential TB services. Home-based sample collection facilities and sending reports using mobile technology can also be emphasized as potential solutions to minimize disruption of TB services during the future pandemic period. Lessons from the current pandemic can also inform the country’s preparedness for future epidemics, and should involve ‘catch-up’ plans for screening, evaluation and treatment of TB during an epidemic.

## Figures and Tables

**Table 1:** Demographic data of the study Upazila and Upazila Health Complex, 2021 Bangladesh [15,16,17,18,19,20,21].

Upazilla Demographic Data, 2021				Upazila Health Complex Demographic Data, 2021				
Name	Total Area in sq. km	Total Population	Population Density per sq. km	Name	No. of Beds	OPD Visits	Emergency Visits	Admissions
Sirajdikhan	180.19	288,107	1599	Sirajdikhan Upazila Complex	50	95,315	13,479	3834
Kaliganj	271.51	239,660	1101	Kaliganj Upazila Health Complex	50	106,042	10,326	6011
Savar	280.12	1,442,885	4948	Savar Upazila Health Complex	50	123,715	8311	4351
Singair	217.56	287,451	-	Singair Upazila Health Complex	50	66,817	6177	6687
Harirampur	245.42	139,318	698	Harirampur Upazila Health Complex	50	62,179	2939	2688
Rupganj	234.76	403,629	2291	Rupganj Upazila Health Complex	50	47,745	5020	2463

OPD = outpatient care department; sq. km = square kilometer.

**Table 2:** Background characteristics of service providers and recipients, 2021, Dhaka division, Bangladesh.

Characteristics	Service Recipients	Service Providers
	n (%)	n (%)
**Total**	**180 (100)**	**18 (100)**
**Age in year**		
Mean ± SD	43 ± 17	39 ± 9.5
**Sex**		
Male	121 (67)	15 (83)
**Residence**		
Urban	62 (34)	-
Rural	118 (66)	-
**Type of family**		
Nuclear	119 (66)	-
Joint	61 (34)	-
**Occupation**		
Homemaker	43 (24)	-
Daily wager	25 (14)	-
Employee	23 (13)	-
Laborer	22 (12)	-
Business	19 (11)	-
Retired	20 (11)	-
Other	18 (10)	-
Student	10 (6)	-
**Designation**		
Laboratory staffs	-	5 (28)
TLCA	-	5 (28)
Medical officer	-	4 (22)
MODC	-	2 (11)
DOTs service provider	-	2 (11)
**Experience in working TB control program (months)**		
Mean ± SD	-	8.6 ± 7.6
**Persons working in TB control program**		
Mean ± SD	-	3 ± 0.6
**Ever infected with COVID-19**		
Yes	-	9 (50)
**Days of taking leave due to being infected with COVID-19 (n = 9)**		
Mean ± SD	-	12 ± 4.7

Note: DOTs (Directly Observed Treatment Short-course); MODC (medical officer disease control); SD, (standard deviation); TLCA (Tuberculosis and Leprosy Control Assistant).

**Table 3:** Comparison of TB service delivery indicators between before and during the COVID-19 pandemic, March 2019 to February 2021, Dhaka division, Bangladesh.

Service Indicators	Total Number		% Reduction
	Before COVID-19 (19 March–20 February)	During COVID-19 (20 March–21 February)	
Presumptive TB patients’ notifications	30,244	20,761	31
Sputum microscopy tests	30,244	19,204	36
Patients tested positive by sputum microscopy	1929	1311	32
Registered adult PTB cases	3677	3091	16

**Table 4:** Types of reported barriers faced by the service providers in the DOTs clinic while providing service during COVID-19, 2021, Dhaka division, Bangladesh (n = 18).

	**Frequency (%)**
**Faced any challenges during providing services due to COVID-19**	
Yes	16 (89)
Challenges told by them due to CVOID-19 pandemic * (n = 16)	
Lack of manpower	16 (100)
Increased workload	15 (94)
Interruption in the regular follow-up of the patient	10 (63)
Interruption in the TB diagnosis	10 (63)
Interruption in the drug supply	4 (25)
**Any adaptation in the DOTs corner due to COVID-19**	
Yes	18 (100)
Type of adaptation in the DOTs corner * (n = 18)	
Behavioral adaptation	17 (94)
Management of TB patients	17 (94)
TB screening management	17 (94)
TB diagnosis	14 (78)
Follow-up	8 (44)
**Having any barrier in contact screening performing**	
Yes	16 (89)
Type of barriers in contact screening told by them * (n = 16)	
Lack of workforce	15 (94)
Lack of patient compliance	16 (100)
**Any barrier in TB diagnosis due to COVID-19**	
Yes	16 (89)
Type of barriers in TB diagnosis * (n = 16)	
Interruption in the sample collection	16 (100)
Reduced visits of the patient to the facility due to COVID-19 panic	16 (100)
Interruption in the performing test	10 (62)
Interruption in the report delivery	7 (44)
Type of barriers in TB treatment * (n = 16)	
Lack of patient compliance	16 (100)
Reduced number of visits of the patient to the facility due to COVID-19 panic	14 (87)
Co-infection with COVID-19	9 (56)
**Any barrier in TB patients’ follow-up due to COVID-19**	
Yes	15 (83)
Type of barriers in TB patients’ follow-up * (n = 15)	
Follow-up visit cancellation	10 (67)
Reduced number of visits	13 (87)
Others	10 (67)

* Multiple responses.

## Data Availability

Not applicable.

## Supplementary Materials

The following supporting information can be downloaded at: https://www.mdpi.com/article/10.3390/tropicalmed7110385/s1.
