# Supplementary material for: The Effect of the COVID-19 Pandemic on Pulmonary Tuberculosis Control in the Selected Upazila Health Complexes of Dhaka Division, Bangladesh
Source: Trop Med Infect Dis. 2022 Nov 17;7(11):385. doi: 10.3390/tropicalmed7110385 (PMC9695409; doi:10.3390/tropicalmed7110385)
Supplement: Supplementary file 1 [file tropicalmed-07-00385-s001.zip › tropicalmed-2034417-supplementary.pdf]

Tropical Medicine and Infectious Disease

Supplementary Materials for The Effect of the COVID-19 Pandemic on Pulmonary Tuberculosis Control in the Selected Upazila Health Complexes of Dhaka Division, Bangladesh

Personal Information	
Facility Name	
Facility address	

Document Review Checklist

DOTS corner, Upazila Health Complex

Reviewed Year: 2020–2021

	Presumptive TB Patients' Notifications	Sputum Microscopy Tests	Patients Tested Positive by Sputum Microscopy	Registered Adult PTB Cases
March 2019				
April 2019				
May 2019				
June 2019				
July 2019				
August 2019				
September 2019				
October 2019				
November 2019				
December 2019				
January 2020				
February 2020				
March 2020				
April 2020				
May 2020				
June 2020				
July 2020				
August 2020				
September 2020				
October 2020				
November 2020				
December 2020				
January 2021				
February 2021				

Personal Information

Name of the respondent	
Facility Name	
Facility address	
Respondent Mobile number	
Time of interview	

Questionnaire for the Health Care Providers Associated with PTB Control

Question	Coding
Age in Years	
Gender	1 = Male<br>2 = Female
Designation	1 = Medical officer<br>2 =DOTS service provider<br>3 = Laboratory staffs<br>4 = TLCA<br>5 = MODC
How many months duration of experience in TB control program?	In months
How many persons usually worked in the TB control program?	
Had they ever been infected with COVID-19?	1 = Yes<br>2 = No
If yes, how many days you took leave due to COVID-19?	-------------
Have you faced any challenges due to the COVID-19 pandemic?	1 = Yes<br>2 = No
If Yes, what were the challenges?	1 = Lack of manpower<br>2 = Increased workload<br>3 = Interruption in the regular follow-up of the TB patient<br>4 = Interruption in the TB diagnosis<br>5 = Interruption in the drug supply<br>6 = Others
Was there any adaptation in the DOTs clinic due to COVID-19?	1 = Yes<br>2 = No
If yes, what was the adaptation?	1 = Behavioral adaptation<br>2 = Management of TB patients<br>3 = TB screening management<br>4 =TB diagnosis<br>5 = Follow-up
Did you perform contact screening regularly?	1 = Yes<br>2 = No
If yes, what were the barriers?	1 = Lack of workforce<br>2 = Lack of patient compliance
Have you faced any barriers in the diagnosis of PTB?	1 = Yes<br>2 = No
If yes, what are they?	1 = Interruption in the sample collection<br>2 = Reduced visits of the patient to the facility due to COVID-19 panic<br>3 = Interruption in the performing test<br>4 = Interruption in the report delivery
Have you faced any barriers in the treatment of TB?	1 = Yes

	2 = No
If yes, what are they?	1 = Lack of patient compliance<br>2 = Reduced number of visits of the patient to the facility due to COVID-19 panic<br>3 = Co-infection with COVID-19
Have you faced any barriers in the follow-up of TB?	1 = Yes<br>2 = No
If yes, what are they?	1 = Follow-up visit cancellation<br>2 = Reduced number of visits<br>3 = Others

Questionnaire for the PTB Patients

Question	Coding
Age in Years	
Gender	1 = Male<br>2 = Female
Residence	1 = Urban<br>2 = Rural
Type of family	1 = Nuclear<br>2 = Joint
Occupation	1 = Homemaker<br>2 = Daily wager<br>3 = Employee<br>4 = Laborer<br>5 = Business<br>6 = Retired<br>7 = Other<br>8 = Student
Have you faced any barriers to coming to seek care at the DOTS corner since COVID- 19?	1= Yes<br>2= No
If yes, what were the barriers for you since COVID-19?	1 = Transport shortage/transportation-related difficulties<br>2 = Fear of getting infected with SARS-COV2 infection<br>3 = Reduced income<br>4 = Long waiting times to get a consultation<br>5 = Shortage of DOTs providers
Are you dissatisfied with the treatment you are receiving during the pandemic?	1 = Yes<br>2 = No
If yes, what were the reasons for the dissatisfaction	1 = Overwrowding in the healthcare facility than usual<br>2 = Unable to get a consultation in time<br>3 = Long waiting times to do any investigation<br>4 = Insufficinet drug supply<br>5 = Reduced the number of follow up<br>6 = Shortage in the diagnostic services
